# 

**Published:** 1857-06

**Authors:** 


VOL. VI. NEW SERIES. No. 6.

THE

### NORTH-WESTERN

### MEDICAL AND SURGICAL

### JOURNAL.

PUBLISHED MONTHLY.

AT TWO DOLLARS PER ANNUM, IN ADVANCE.

EDITED BY

N. S. DAVIS, M.D.,

PROFESSOR OF PRINCIPLES AND PRACTICE OF MEDICINE AND CLINICAL MEDICINE IN RUSH MEDICAL COLLEGE; ONE OF THE PHYSICIANS TO THE MERCY HOSPITAL; FELLOW OF THE COLLEGE OF PHYSICIANS AND SURGEONS, NEW YORK; MEMBER OF THE MEDICAL SOCIETY OF THE STATE OF NEW YORK, AND OF THE STATE OF ILLINOIS; MEMBER OF THE AMERICAN MEDICAL ASSOCIATION, &C. &C.

JUNE, 1857.

CHICAGO:

ROBERT FERGUS, PRINTER,

189 LAKE STREET.

VOL. XIV. WHOLE SERIES. No. 6.

